# RTL551 Treatment of EAE Reduces CD226 and T-bet+ CD4 T Cells in Periphery and Prevents Infiltration of T-bet+ IL-17, IFN-γ Producing T Cells into CNS

**DOI:** 10.1371/journal.pone.0021868

**Published:** 2011-07-05

**Authors:** Sushmita Sinha, Lisa M. Miller, Sandhya Subramanian, Gregory G. Burrows, Arthur A. Vandenbark, Halina Offner

**Affiliations:** 1 Neuroimmunology Research, Veterans Affairs Medical Center, Portland, Oregon, United States of America; 2 Department of Neurology, Oregon Health & Science University, Portland, Oregon, United States of America; 3 Department of Biochemistry and Molecular Biology, Oregon Health & Science University, Portland, Oregon, United States of America; 4 Research Service, Department of Veterans Affairs Medical Center, Portland, Oregon, United States of America; 5 Department of Molecular Microbiology & Immunology, Oregon Health & Science University, Portland, Oregon, United States of America; 6 Department of Anesthesiology and Perioperative Medicine, Oregon Health & Science University, Portland, Oregon, United States of America; Charité Universitaetsmedizin Berlin, Germany

## Abstract

Recombinant T cell receptor ligands (RTLs) that target encephalitogenic T-cells can reverse clinical and histological signs of EAE, and are currently in clinical trials for treatment of multiple sclerosis. To evaluate possible regulatory mechanisms, we tested effects of RTL therapy on expression of pathogenic and effector T-cell maturation markers, CD226, T-bet and CD44, by CD4+ Th1 cells early after treatment of MOG-35-55 peptide-induced EAE in C57BL/6 mice. We showed that 1–5 daily injections of RTL551 (two-domain I-A^b^ covalently linked to MOG-35-55 peptide), but not the control RTL550 (“empty” two-domain I-A^b^ without a bound peptide) or Vehicle, reduced clinical signs of EAE, prevented trafficking of cells outside the spleen, significantly reduced the frequency of CD226 and T-bet expressing CD4+ T-cells in blood and inhibited expansion of CD44 expressing CD4+ T-cells in blood and spleen. Concomitantly, RTL551 selectively reduced CNS inflammatory lesions, absolute numbers of CNS infiltrating T-bet expressing CD4+ T-cells and IL-17 and IFN-γ secretion by CNS derived MOG-35-55 reactive cells cultured ex vivo. These novel results demonstrate that a major effect of RTL therapy is to attenuate Th1 specific changes in CD4+ T-cells during EAE and prevent expansion of effector T-cells that mediate clinical signs and CNS inflammation in EAE.

## Introduction

Recombinant T cell receptor ligands (RTLs) reverse clinical and histological signs of EAE in an antigen-specific manner, and are currently in clinical trials for treatment of multiple sclerosis [1], [2], [3]. Our earlier studies showed that treatment with single RTLs can induce a cytokine switch in cognate T-cells that inhibits both target and bystander T-cells [4]. Recently we have shown that RTLs bind to surface receptors on B cells, macrophages and dendritic cells, but not T-cells, through the MHC class II α1β1 moiety of the RTL in an antigenic peptide-independent manner [5]. Antigen specificity in RTL treatment of EAE strongly suggests potential tolerogenic signals being delivered to T-cells following RTL binding with APCs. In fact, our preliminary studies have demonstrated that DR2-derived RTLs could induce changes in cytokine secretion patterns without proliferation in human T-cell clones [6]. Moreover, RTL201 (comprised of the rat RT1B MHC moiety linked to Gp-MBP-72-89 peptide) could induce partial activation of the cognate A1 T-cell hybridoma involving a CD3ζ p23/p21 ratio shift, ZAP-70 phosphorylation, internal calcium mobilization, NFAT activation, and transient IL-2 production [7]. However, the downstream effects of early signaling induced in T-cells by RTLs that could potentially regulate clinical EAE and cause its attenuation remain largely unknown. Considering the fact that CD4 T-cells are initiators of EAE and are drivers of neuro-immune degeneration in CNS [8], this study was designed with the aim of obtaining mechanistic insights by exploring RTL551 (two-domain I-A^b^ covalently linked to MOG-35-55 peptide) effects on CD4+ T-cells in vivo after induction of clinical and histological signs of EAE with MOG-35-55/CFA/Ptx in C57BL/6 mice.

Rapid resolution of EAE after RTL treatment prompted us to study early time points after treatment initiation. Empty RTL550 (“empty” two-domain I-A^b^ without a bound peptide) was used as additional control to provide evidence that irrespective of similar binding of empty RTLs to APCs, the nature of the bound peptide is the determining factor for subsequent signal transduction in immune cells. We showed that a single injection of RTL551, but not the control RTL550, reduced clinical signs of EAE, prevented trafficking of cells outside the spleen, significantly reduced the frequency of CD226 and T-bet expressing CD4+ T-cells in blood and inhibited expansion of CD44 expressing CD4+ T-cells in blood and spleen. Concomitantly, RTL551 selectively reduced absolute numbers of T-bet+CD4+ T-cells and IL-17 and IFN-γ secretion in the CNS. These novel results demonstrate that a major effect of RTL therapy is to attenuate encephalitogenic activity of Th1/Th17+ CD4+ T-cells during EAE and prevent maturation of memory T-cells that mediate clinical signs and CNS inflammation in EAE.

## Materials and Methods

### Animals

C57BL/6 male mice were obtained from Jackson Laboratories (Bar Harbor, ME) at 7–8 wk of age. The mice were housed in the Animal Resource Facility at the Portland Veterans Affairs Medical Center (Portland, OR) in accordance with institutional guidelines. The study was conducted in accordance with National Institutes of Health guidelines for the use of experimental animals, and the protocols were approved by the Institutional Animal Care and Use Committee, protocol # 4508, local database ID # 2313.

### Antigen

Mouse MOG-35-55 peptide (MEVGWYRSPFSRVVHLYRNGK) was synthesized from NeoMPS.

### RTL construction, modification and production

General methods for the design, cloning and expression of RTLs have been described previously [3], [9]. In brief, a series of murine MHC class II I-A^b^-derived single chain beta-1/alpha-1 recombinant T-cell receptor ligands (“RTLs”), termed rI-A^b^ (RTL550), were constructed by sequential site-directed mutagenesis of rI-A^q^ RTLs. The progenitor rI-A^q^ RTLs were constructed using mRNA isolated from the splenocytes of DBA1/LacJ mice using an Oligotex Direct mRNA mini kit (Qiagen, Inc., Valencia, CA). cDNA of the antigen binding/TCR recognition domain of murine I-A^q^ MHC class II β1 and α1 chains was derived from mRNA using two pairs of PCR primers. The two chains were sequentially linked by a 5 amino acid linker (GGQDD) in a two-step PCR reaction with NcoI and XhoI restriction sites being added to the amino terminus of the β1 chain and to the carboxyl terminus of the α1 chain respectively, to create rI-A^q^ (RTL560). “Empty” rI-A^b^ (RTL550) was constructed by sequential site-directed mutagenesis using “empty” rIA^q^ ((RTL560) as template. The 22 site-directed mutations required were inserted using eight primers containing the desired sequence changes and 4 consecutive mutagenesis cycles (QuikChange Multi site-directed mutagenesis kit, Stratagene, Inc). Additional sequence encoding murine MOG-35-55 peptide (MEVGWYRSPFSRVVHLYRNGK) and a flexible linker (GSGSGSGSGSGSGS) was added to the 5′ end of the β1 domain of rI-A^b^ RTL550 to form rI-A^b^/MOG (RTL551). The rI-A^b^/MOG insert was ligated into pET21d(+) vector and transformed into a Nova blue *E. coli* host (Novagen, Inc., Madison, WI) for positive colony selection and sequence verification, and rI-A^b^/MOG plasmid constructs were then transformed into the *E. coli* strain BL21(DE3) expression host (Novagen, Inc., Madison, WI). Protein purification was as previously described [9], [10] with a 30 to 40 mg yield of purified protein per liter of bacterial cell culture.

### Induction of active EAE and treatment with RTLs

C57BL/6 mice were inoculated in flanks with 0.2 ml of emulsion containing 200 µg of MOG-35-55 peptide and an equal volume of CFA containing 2 mg/ml of heat killed *Mycobacterium tuberculosis*. Mice were also injected with 75 ng and 200 ng pertussis toxin (Ptx) intraperitoneally on day 0 and 2 relative to immunization. The mice were assessed for signs of EAE according to the following scale: 0, normal; 1, limp tail or mild hind-limb weakness; 2, moderate hind-limb weakness or mild ataxia; 3, moderately severe hind-limb weakness; 4, severe hind-limb weakness or mild fore-limb weakness or moderate ataxia; 5, paraplegia with no more than moderate fore-limb weakness; and 6, paraplegia with severe fore-limb weakness or severe ataxia or moribund condition. At the onset of clinical signs of EAE (days 12–14 when the clinical scores were≥2), the mice were divided into three groups and treated i.v. with 100 µl buffer as Vehicle-control, 100 µl of 1 mg/ml RTL551 (containing MOG-35-55 peptide) or 100 µl of 1 mg/ml RTL550 (empty) daily for 5 days. Mice were monitored for changes in disease score until they were euthanized for ex vivo analyses.

### Flow cytometry

Four-color (fluoresceinisothiocyanate, phycoerythrin, propidium iodide, allophycocyanin) fluorescence flow cytometry analyses were performed to determine the phenotypes of cells following standard antibody staining procedures. At indicated time points, cardiac blood was collected in EDTA followed by perfusion of the mice with 1xPBS. For splenocytes, single cell suspensions of spleens from vehicle, RTL550 and RTL551 treatment groups were prepared by homogenizing the tissue through a fine mesh screen. Blood cells were pelleted after lysis of red cells followed by washing twice with RPMI. Mononuclear cells from the CNS were isolated by Percoll gradient centrifugation as described [11]. Cells from spleen, blood and CNS were resuspended in staining medium (5% BSA, 1X PBS and 0.02% sodium azide) for FACS staining. For intracellular cytokine determination, cells were stimulated with leukocyte activation cocktail (BD PharMingen, San Diego, CA ) for 6 hrs prior to surface staining. All antibodies were purchased from BD PharMingen (San Diego, CA) or eBioscience (San Diego, CA) unless otherwise indicated. Cells were stained with the combination of following antibodies: CD4, CD8, CD226, CD44. Intracellular staining of T-bet (Biolegend; San Diego, CA), IL17 and IFNγ (BD PharMingen, San Diego, CA) was completed following overnight incubation in fixation/permeabilization buffer (eBiosciences) and incubation with respective primary antibodies for 30 min. After staining, cells were washed with staining medium and analyzed immediately with a FACS Calibur using FCS express (Los Angeles, CA) software. Data represent 20,000 live gated events. Absolute numbers of cells were calculated from live-gated CNS cells.

### Cytokine determination by multiplex luminex kit

Mononuclear cells from the CNS of individual mice were cultured at 200,000 cells/well in a 96-well round-bottom culture plate in stimulation medium (RPMI, 1% sodium pyruvate, 1% L-glutamine, 0.4% 2-β-mercaptoethanol, 2% FBS) with 25 µg/ml MOG-35-55 peptide for 48 h. Supernatants were then harvested and stored at −80°C until testing for cytokines. Culture supernatants were assessed for cytokine levels using a Luminex Bio-Plex mouse cytokine assay kit (Bio-Rad Laboratories, Inc., Richmond, CA) following manufacturer's instructions. The following cytokines were determined in a single assay in three separate experiments: IFN-γ, TNF-α, IL-2, IL-6, IL-13 and IL-17.

### Histopathology

Intact spinal cords were removed from 4 mice in each group at indicated time points of clinical disease and fixed in 10% Formalin for 48 h. All the mice were perfused with PBS prior to organ collection. The spinal cords were dissected after fixation and embedded in paraffin before sectioning. The sections were stained with H&E to assess inflammatory lesions, and analyzed by light microscopy.

### Statistical analysis

Statistical differences between disease scores of vehicle, RTL550 and RTL551 treatment groups were determined by Mann-Whitney U test. Differences in percentages of cells expressing various cell surface markers, splenocyte numbers and cytokine responses were evaluated using Student's *t* test. P values≤0.05 were considered significant.

## Results

### Clinical EAE scores are significantly reduced in mice treated with RTL551

**Figure 1:**
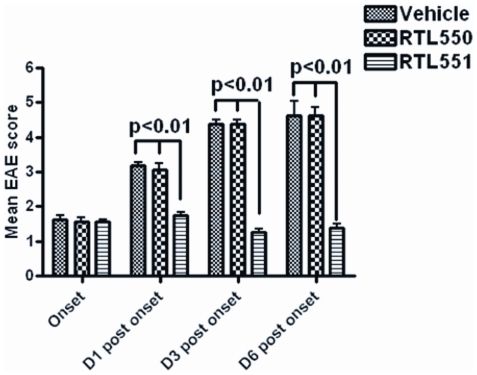
EAE Mice treated with RTL551 at onset have significantly reduced clinical scores as compared to vehicle or RTL550 treated mice. Male C57BL/6 mice were immunized with MOG-35-55/CFA/Ptx. Mice were scored for clinical signs of EAE as outlined in *Materials and Methods*. Data presented are the mean±SD disease scores of 8–10 mice per group. Significant differences between the groups were determined using Mann Whitney *U* test.

Treatment of EAE mice with vehicle, RTL550 or RTL551 was initiated at onset of clinical signs of disease followed by 4 additional daily injections. Clinical scores of EAE on the days of ex vivo procedures are shown in Fig. 1. For all the results, Days (D)1, 3 and 6 post-onset indicates 1, 3 and 5 treatments respectively. EAE progression was completely halted in the mice as early as one RTL551 treatment and the RTL551 treated group had significantly reduced clinical disease scores at all the time points as compared to control groups (Vehicle and RTL550) of mice which showed worsening disease scores over time (Fig. 1).

### RTL551 prevents migration of cells from spleen of EAE mice

**Figure 2:**
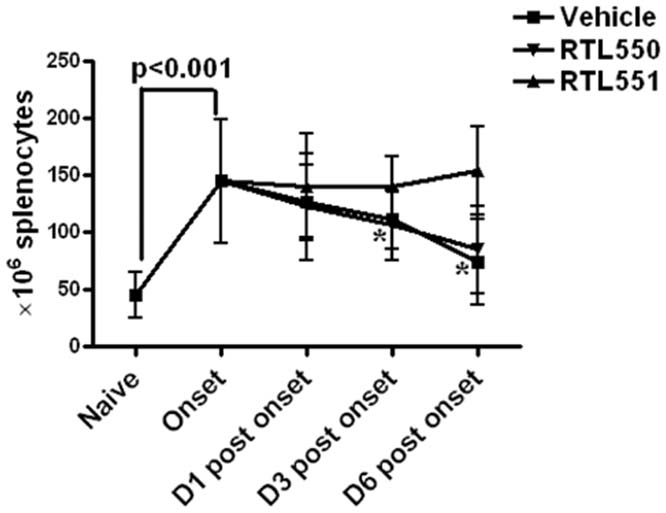
RTL551 prevents migration of cells outside spleen. Splenocytes were counted from naïve mice and mice at indicated time points after EAE induction. As compared to naïve mice, spleen cell numbers increased after immunization and at onset of EAE followed by gradual decline with EAE progression in control groups of mice, possibly due to migration of cells outside the spleen towards the CNS. This trafficking of cells outside the spleen is prevented after RTL551 treatment. Data presented are the mean±SD of 8 mice per group except for naïve which had 5 mice. Significant differences between the groups (p≤0.05) were determined using Student's *t* test and are indicated by an asterisk.

At onset of clinical signs of EAE prior to treatment, there was a significant 3X increase in the splenocyte numbers in mice immunized with MOG-35-55/CFA/Ptx (cell number in naïve vs. EAE mice, 45.5±19.9 vs. 144.8±54.4, Fig. 2). In the acute phase of EAE, cells are still trafficking from the periphery into the CNS, and as EAE progressed, there was a gradual decline in splenocyte numbers in Vehicle and RTL550 treated mice (Fig. 2), suggesting the migration of cells outside the spleen possibly towards the CNS. Interestingly, RTL551-treated mice retained the increased levels of cells in the spleen throughout the observation period.

### RTL551 treated mice had reduced frequency of CD4+ T-cells in the blood at early time points

**Figure 3:**
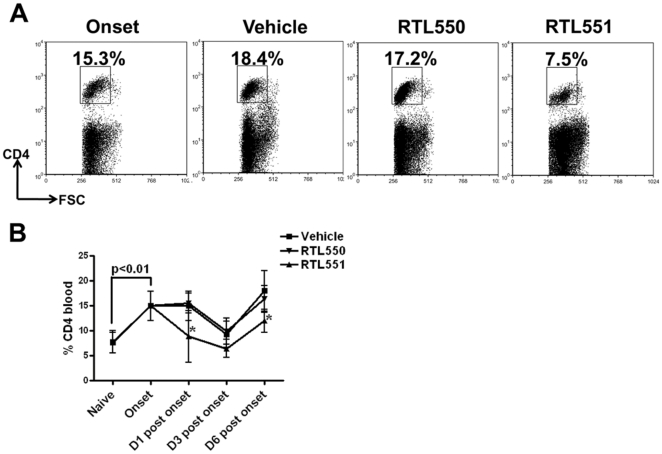
Frequency of CD4+ T-cells is significantly reduced in the blood of mice treated with RTL551. Mice were euthanized at the indicated time points after onset and isolated blood cells were lysed, stained with fluorescent antibodies and analyzed by flow cytometry. Representative dot plot for CD4 staining in blood on D1 is shown in panel A. Sequential changes in CD4+ T-cell frequencies in the blood of mice with EAE are shown in panel B. Data in panel B are mean±SD values from 8 mice per group except for naïve which had 5 mice. Significant differences between the groups (p≤0.05) were determined using Student's *t* test and are indicated by an asterisk.

As compared to naïve mice, EAE mice had a significantly increased percentage of CD4+ T-cells in the blood at onset of clinical signs (naïve vs. onset, 7.7±2.0 vs. 14.9±2.9) and CD4+ T-cell percentages remained significantly higher D1 post onset in Vehicle (14.9±5.6) and RTL550 (15.5±2.0) treated mice compared to RTL551 (8.8±5.9) treated mice (Fig. 3A & B). During this initial phase of EAE, the increase in CD4+ T-cells in the blood corresponds with increases of encephalitogenic CD4+ T-cells in the CNS [12] and thus might indicate their migration from spleen towards the CNS. Percentages of CD4+ T-cells in blood decreased in all treatment groups on D3 post onset, followed by increases in all groups on D6 post onset (Fig. 3B). Of importance, the CD4+ T-cell percentages in the blood of RTL551 treated mice remained significantly lower than Vehicle and RTL550 treated mice at the D6 post onset time point (Fig. 3B).

### RTL551 treatment causes reduction in percentages of CD226 and T-bet expressing CD4+ T-cells in the blood during the early stages of EAE

**Figure 4:**
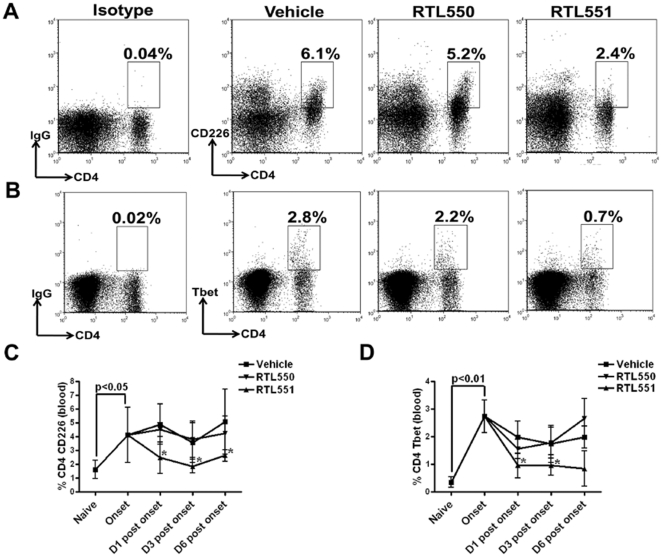
Treatment with RTL551 significantly reduces the percentages of CD226 and T-bet expressing CD4+ T-cells compared to treatment with Vehicle or RTL550 in the blood of mice with EAE early after treatment initiation. Representative dot plots for CD226 and T-bet staining, respectively, on CD4 T-cells in blood are shown in panels A & B. Sequential changes in the expression of CD226 and T-bet on CD4 T-cells in the blood of mice from the three groups during EAE are shown in panels C & D. Data in panels C & D are mean±SD values from 8 mice per group except for naïve which had 5 mice. Significant differences between the groups (p≤0.05) were determined using Student's *t* test and are indicated by an asterisk.

CD226 and T-bet are two key Th1-associated markers that have been linked to the encephalitogenic activity of CD4+ T-cells, and it was thus of interest to evaluate the effects of RTL551 on expression of these markers during treatment of mice with EAE. Concomitant with the increase in CD4+ T-cells in the blood at EAE onset, there were significantly elevated levels of CD226 (naïve vs. EAE onset, 1.6±0.7% vs. 4.1±0.1%) and T-bet (naïve vs. EAE onset, 0.3±0.2% vs. 2.7±0.6%) in the CD4+ T-cells (Fig. 4), thus illustrating the temporal association of these two Th1 markers with manifestation of EAE clinical signs. Of importance, a single injection of RTL551 significantly reduced the percentages of CD4+ CD226 (RTL551 D1 post onset, 2.4±1.1%, Fig. 4C) and T-bet (RTL551 D1 post onset, 1.0±0.5%, Fig. 4D) expressing T-cells in the blood compared to Vehicle or RTL550-treated mice. While the control groups with EAE had consistently elevated levels of CD226 and T-bet expressing CD4+ T-cells in the blood throughout the observed time points (Fig. 4C & D, respectively), mice treated with RTL551 had markedly reduced percentages of these pathogenic CD4+ T-cells.

### RTL551 prevents expansion of effector CD4+ T-cells in the periphery

**Figure 5:**
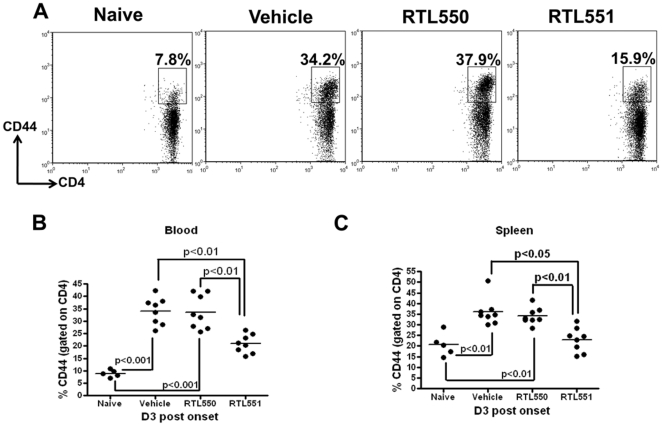
Treatment of EAE mice with RTL551 inhibits expansion of CD44 expressing CD4+ T-cells in the periphery compared to treatment with Vehicle or RTL550. Splenocytes and lysed blood cells were stained with respective antibodies and subjected to flow cytometry. Dot plot for CD44 staining on blood CD4+ T-cells on day 3 post onset is shown in panel A. Cells were gated on live CD4+ cells for this analysis. Values in panels B & C are representative of 8 mice per group on D3 after onset of clinical signs of EAE except for naïve which had 5 mice. Significant differences between the groups (p≤0.05) were determined using Student's *t* test and are indicated by brackets.

CD44 expressing CD4+ T-cells are increased in the spleen and CNS of mice with EAE [13], and it was thus of interest to evaluate effects of RTL551 treatment on this population of activated effector cells during EAE (representative dot plots for CD44 staining on blood CD4+ T-cells on D3 post onset are shown in Fig 5A). The percentage of CD4+ cells expressing CD44 was significantly increased in EAE control mice vs. naïve mice on D3 post EAE onset both in blood (naïve vs. Vehicle & RTL550, 8.9±1.5% vs. 34.4±6.1% & 34.8±7.6%, respectively, Fig. 5B) and spleen (naïve vs. Vehicle and RTL550, 20.6±5.4% vs. 36.2±7.5% & 33.8±4.5% respectively, Fig. 5C). Treatment of EAE mice with RTL551 significantly reduced the percentages of CD44 expressing CD4+ T-cells after 3 injections in the blood (RTL551 D3 post onset, 21.2±4.3%; Fig. 5B) and spleen (RTL551 D3 post onset, 24.7±4.4%, Fig. 5C). The concomitant reduction of CD44 expressing CD4+ T-cells in blood and spleen of RTL551 treated mice on D3 suggests that the reduced percentage in blood was not due simply to sequestration of these cells in the spleen, and might, rather, implicate the ability of RTL551 to prevent the expansion of CD44 expressing CD4+ T-cells in the spleen. In CNS, 100% of CD4+ T-cells were also CD44+ in mice with EAE (not shown).

### Reduction in IL-17 and IFN-γ producing cells in the spleen after RTL551 treatment

**Figure 6:**
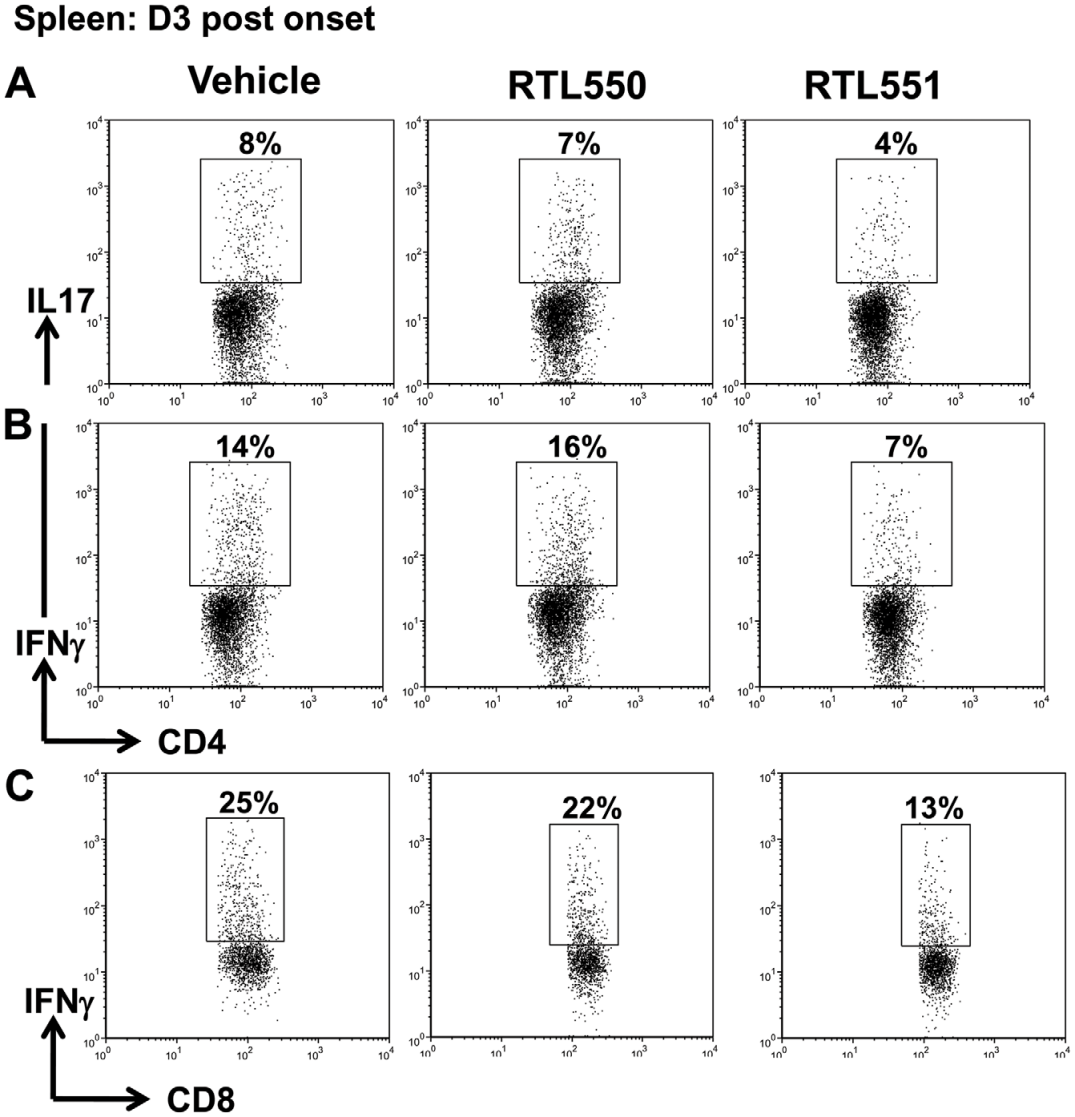
RTL551 treated mice had reduced frequencies of IL-17 and IFNγ secreting cells in the spleen D3 post onset. Splenocytes were stimulated with leukocyte activation cocktail and were stained with respective antibodies and subjected to flow cytometry. Representative dot plots for IL-17 and IFN-γ staining (respectively) on CD4+ T-cells are shown in panels A & B respectively. Cells were gated on CD4+ cells for this analysis. IL-17 staining on gated CD8+ T-cells is shown in panel C. Data are representative of 3 mice per group.

In order to study effector functions of the cells retained in the spleen of RTL551 treated mice, intracellular staining for IL-17 and IFN-γ was performed (Fig. 6). While D1 post onset spleens of the three groups of mice had similar frequencies of IL-17 and IFN-γ producing cells (data not shown), significantly reduced frequencies of IL-17 (D3 post onset, vehicle and RTL550 vs. RTL551, 8±1.4 and 7.3±0.4; vehicle & RTL550 vs. RTL551, p<0.05) and IFN-γ (D3 post onset, vehicle and RTL550 vs. RTL551, 15±1.2 and 15.7±1 vs. 7.9±1.2; vehicle & RTL550 vs. RTL551, p<0.05) producing CD4+ T cells were present in the spleens of RTL551 treated mice (Fig. 6A & B). These observations were also expanded to CD8+ T-cells to study whether inhibition of Th1 CD4+ T cells in RTL551 treated mice influenced effector functions of CD8+ T cells, too. Although we could not detect any IL-17 secreting CD8+ T cells (data not shown), IFN-γ producing CD8 T cells were reduced in the splenocytes of RTL551 treated mice on D3 post onset although the difference was not significant (D3 post onset, vehicle and RTL550 vs. RTL551, 23±2.8 and 21±3 vs. 9.3±4.8; vehicle & RTL550 vs. RTL551, p = 0.07, Fig. 6C).

### Rapid resolution of CNS inflammation in mice treated with RTL551

**Figure 7:**
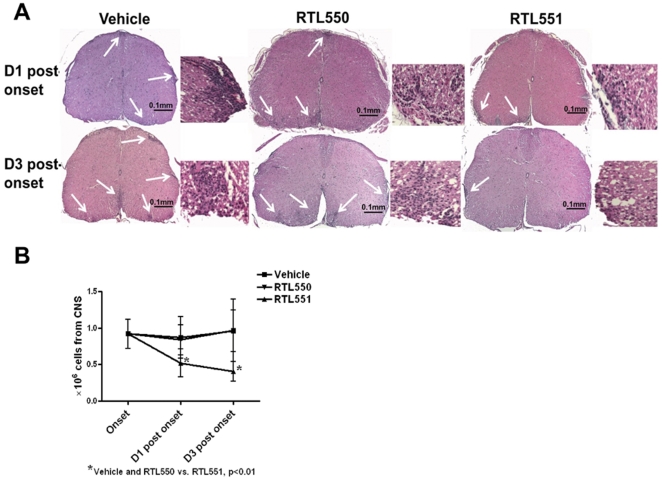
RTL551 treated mice have less infiltration of immune cells in the CNS. Panel A represents H&E staining on paraffin embedded spinal cord sections from Vehicle, RTL550 or RTL551 treated C57BL/6 mice at indicated time points after EAE onset. Spinal cords from control groups of mice (Panel A) showed dense mononuclear infiltration (arrows) and sections from RTL551 treated mice showed reduction in inflammatory cells. Magnification: A, 5X; insets, 20X. Panel B is the mean±SD values of cells isolated from the CNS of mice from the three groups (8 mice per group) at indicated time points. Significant differences between the groups (p≤0.05) were determined using Student's *t* test and are indicated by an asterisk.

Previously we reported a marked reduction of infiltrating cells and a complete absence of inflammation in the CNS after completing treatment of EAE with RTL551 [3]. Here we now evaluate these factors in the CNS at early time points after RTL551 treatment to look for temporal changes. While the control groups of mice had the expected increases in CNS inflammatory lesions, RTL551 treated mice had a striking reduction in CNS inflammation even after a single treatment (D1 post onset, Fig. 7A). Inflammation continued to resolve in RTL551 treated mice (D3 post onset, Fig 7A) and as published earlier, there was complete lack of inflammatory cells in the spinal cords by D6 post onset (data not shown). Correspondingly, there were significantly fewer inflammatory cells isolated from the CNS of RTL551-treated mice during these early time points (Fig. 7B). CNS analysis was limited to 3 days post onset due to complete resolution of inflammation in the CNS of RTL treated mice by D6 post onset.

### Selective reduction in recruitment of T-bet expressing CD4+ T-cells into the CNS of mice treated with RTL551

**Figure 8:**
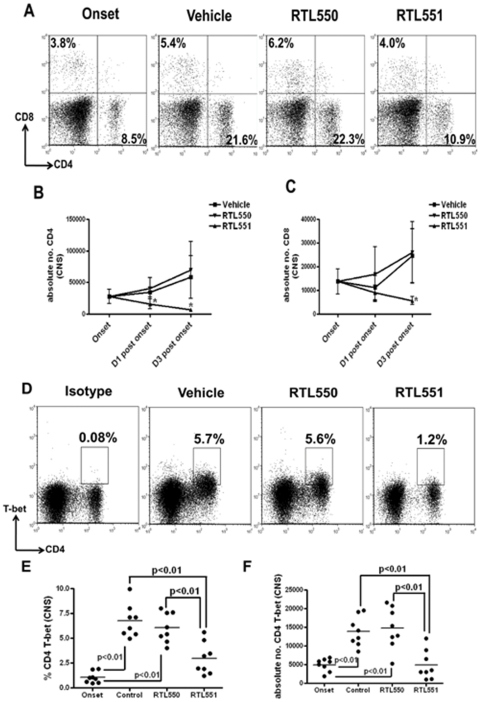
Selective reduction of T-bet expressing CD4 T-cells from the CNS of EAE mice treated with RTL551. T-bet expressing CD4+ T-cells were increased in the CNS of control groups of mice with EAE when compared to onset. One injection of RTL551 induced significant reduction and recruitment of CD4+ T-cells into the CNS. Representative dot plots for CD4+ and T-bet staining on the cells isolated from CNS of mice are shown in panels A & D. Sequential changes in mean±SD absolute numbers of CD4+ and CD8+ cells in the CNS of control and RTL551 treated mice are shown in panels B & C. Percentages are demonstrated in panel E and panel F shows absolute numbers of T-bet expressing CD4+ T-cells in the CNS of mice D1 post onset. There were 8 mice per group and significant differences between the groups (p≤0.05) were determined using Student's *t* test and are indicated by an asterisk (panels C and D) or by brackets (panels E and F).

Immuno-phenotyping of cells isolated from the CNS at indicated time points after onset of EAE revealed that absolute numbers of CD4+ T-cells recovered from the CNS increased with time in the control groups (Fig. 8A & B). However, a single RTL551 treatment resulted in reduced percentages as well as significantly reduced absolute numbers (Fig. 8 A & B) of CD4+ T-cells obtained from the CNS (absolute number of D1 vehicle & RTL550 vs. RTL551, 34,906±6,927 & 45,599±14,318 vs. 16,023 vs. 7,845) and these differences were even more pronounced on D3 (Fig. 8B). A similar pattern of RTL551 inhibition of infiltrating CD8+ T-cells was also observed on D3 but not on D1 post onset of EAE (Fig 8C). Of interest, the highest expression of T-bet on CD4+ CNS T-cells was in the control groups at D1 post onset (vehicle & RTL550, 7.2±1.7% & 5.9±1.5%, respectively; Fig. 8D &, E), a significant increase as compared to onset (1.2±0.6%, p<0.001, Fig. 8E). However, the percentage (3.4±1.7%, p<0.01, Fig 8E) and absolute number (Fig. 8F) of T-bet expressing CD4+ T-cells recovered on D1 from the CNS of RTL551 treated mice were significantly reduced vs. D1 controls, but were not significantly increased vs. cells recovered at onset. This result suggests that a single RTL551 injection limited the number of infiltrating T-bet expressing CD4+ T-cells into the CNS.

### RTL551 induces early down-regulation of IL-17 and IFN-γ in the CNS

**Figure 9:**
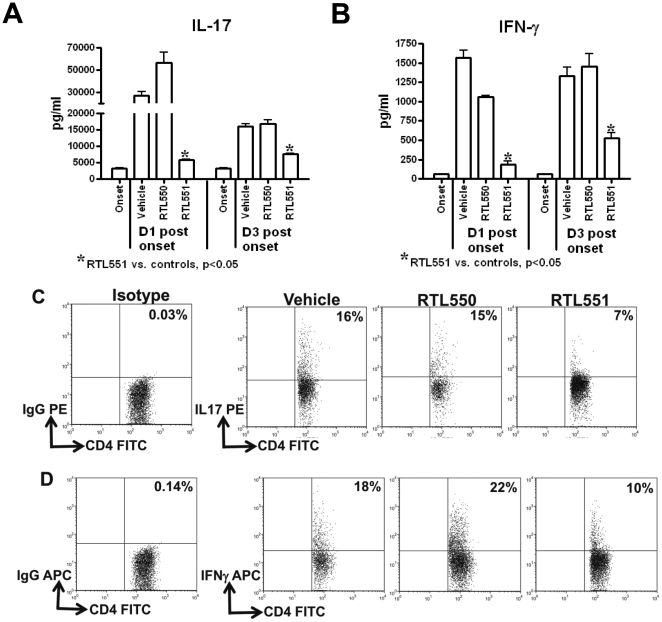
IL-17 and IFN-γ were significantly reduced in the culture supernatants of cells isolated from the CNS of RTL551 treated mice as compared to control groups of mice (panel A & B). Cytokine production was quantified in the supernatants from mononuclear cells isolated from CNS of mice at indicated time points and stimulated with MOG-35-55 peptide in vitro. Supernatants were harvested after 48h of culture and assayed for cytokine production using a Bio-Plex assay kit. Data are presented as the mean±SD of three replicate cultures from pooled cells, and are representative of 4 individual mice. Significant differences between the groups (p≤0.05) were determined using Student's *t* test and are indicated by an asterisk. RTL551 treated mice had reduced frequency of IL-17 and IFN-γ producing CD4 T-cells in the CNS (Fig 9 C & D).

The rapid reduction of inflammatory infiltrates from CNS has always been an intriguing feature of RTL mediated treatment of EAE. An elegant recent study showed that cells expressing the Th17+CD4+ phenotype (particularly) have the potential for initiating immune mediated neuronal dysfunction in mice with EAE [14]. This led us to evaluate the levels of IL-17 and IFN-γ, along with other cytokines, in the CNS during the early phases of RTL551 treatment. During acute EAE, cells isolated from CNS secreted high concentrations of IL-17, the highest levels detected on D1 after onset (Fig. 9). Of paramount importance, reduction in CD4+ T-cells in the CNS of RTL551 treated mice at this time point (Fig. 8B) was associated with a significant reduction in IL-17 secretion in culture supernatants of MOG-35-55 peptide-stimulated CNS cells (Fig. 9A). This was also associated with reduced frequency of IL-17 producing cells in RTL551 treated CNS as suggested by intracellular staining (Fig 9C). This is a key finding that could be linked to RTL-mediated early intervention in the EAE pathogenic process, causing disruption in the inflammatory response and potentially sparing downstream neuronal damage mediated by Th17 cells in the CNS. Considerable, albeit lower, concentrations of IL-17 were still detected in the culture supernatants of CNS cells isolated from D3 post EAE onset mice from the control groups (Fig. 9), with continued inhibition by RTL551 at this time point. Of added interest, the key Th1 cytokine, IFN-γ, was also present in the MOG-35-55 activated culture supernatants in control mice during early EAE (Fig. 9B & D), and was also strongly inhibited in RTL551-treated mice. All the other cytokines evaluated, including TNF-α, IL-2, IL-6 and IL-13, were present at very low concentrations in the MOG-35-55 peptide stimulated CNS cell culture supernatants and were not different between RTL551 treated and control groups.

## Discussion

Previous studies from our laboratory have established the ability of RTL constructs to treat EAE in several different rat and mouse models [2], [3], [4], [15], [16], [17]. In all the models tested, cognate RTL when used to treat EAE at onset induced rapid remission from clinical EAE which is associated with resolution of CNS inflammation. In this study, we explored the mechanism and effect of RTLs specifically on encephalitogenic CD4+ T-cells in the C57BL/6 model of EAE induced with MOG-35-55/CFA/Ptx. Rapid resolution of EAE after RTL551 treatment prompted us to explore immunologic changes at early time points after EAE induction. We established earlier that RTLs bind to antigen presenting cells (APCs) through MHC class II α1β1 moiety and this is independent of covalently bound peptide [5]. Several studies from our group have shown that RTL treatment of EAE is very antigen specific such that RTL551 will not inhibit EAE induced with PLP-139-151 and RTL401 (containing PLP-139-151) is ineffective in MOG-35-55 induced EAE [1], [15]. These facts strongly indicate towards undefined tolerogenic signals being transmitted to CD4+ T-cells subsequent to RTL binding with APCs. Moreover, unlike RTLs with covalently bound peptide, empty RTL constructs have no effect on EAE progression [15], suggesting that empty RTLs bound to APCs are unable to deliver a complete set of tolerogenic signals to T cells. Furthermore, we have shown in the past that equimolar concentration (as cognate RTL) of encephalitogenic peptide administered i.v. at EAE onset had no significant effect on the progression of EAE [15].

The current study presents novel findings concerning cellular effects of RTL551 on antigen experienced CD4+ T-cells. The most important conclusions from this study are: 1) RTL551 treatment sequesters the encephalitogenic T-cells within the spleen; 2) RTL551 selectively affects CD4+ T-cells and causes reduction in CD226 and T-bet expressing CD4+ T-cells in the blood early during EAE; 3) CD44 expressing CD4+ effector T-cells are expanded in the periphery during EAE and RTL551 treatment prevents expansion of these effector T-cells; and 4) All these peripheral events lead to reduced infiltration of T-bet expressing and IL-17 and IFN-γ secreting CD4+ T-cells into the CNS. This study provides significant insights into the early and sequential changes influencing encephalitogenicity of CD4+ T-cells during EAE and the fate of encephalitogenic CD4+ T-cells after RTL551 administration at EAE disease onset.

Mice were evaluated at early time points after onset of clinical signs of EAE and treatment with vehicle, “empty” RTL550 or RTL551 containing MOG peptide to follow the fate of encephalitogenic MOG-35-55 peptide-reactive CD4+ T-cells. At onset of EAE, there was three-fold increase in spleen cell numbers in the mice immunized with MOG/CFA/Ptx as compared to naive mice (Fig. 2). Furthermore, spleen cell numbers steadily decreased with the progression of EAE in control groups of mice, suggesting their migration out of the spleen towards the CNS. However RTL551 treated mice maintained increased spleen cell numbers at all the time points tested. Considering that spleen cell numbers were higher in RTL551 treated mice at D1, D3 and D6 post onset and that the percentages of major cellular subtypes in the spleen were not different at any of the time points between groups, it is apparent that absolute numbers of each of these cellular subtypes were higher in the spleen of RTL551 treated mice. Therefore, further evidence for any specific cellular subtype affected by RTL551 treatment was sought in the blood of EAE mice treated at onset with vehicle, RTL550 or RTL551. We found a significant increase (as compared to naïve mice) in CD4+ T-cells in the blood of control groups of mice at onset and on D1 after onset (Fig. 3). During this early post-priming time point in EAE, cells are making their way into the CNS, particularly CD4+ T-cells which are the major initiators of EAE [8]. A significant decrease in the frequency of CD4+ T-cells in the blood after one RTL551 injection provides evidence for a major crucial intervention in EAE pathogenesis caused by RTL551. We strongly believe that RTL551 is affecting the antigen experienced CD4+ T-cell in the blood because: 1) RTL551 injections given to naïve mice had no effect on CD4+ T-cells in any organ including blood (data not shown); and 2) empty RTL550 had no effect on the frequencies of CD4+ T-cells in the blood of EAE mice (Fig. 2). We observed interesting kinetics for expression of two key Th1 cell markers, CD226 and T-bet, on CD4+ T-cells in the blood of control groups of mice (Fig. 4). Recently, CD226 has been shown to be present selectively on differentiated Th1 cells and while it is constitutively expressed by CD8+ T-cells, CD226 is upregulated on CD4+ T-cells upon activation [18], [19], [20]. Nonsynonymous single nucleotide polymorphism rs763361/Gly307Ser in exon 7 of CD226 has been associated with multiple autoimmune diseases including T1D, MS and possibly AITD and RA [19]. In T-cells CD226 is functional only upon activation and it physically associates with LFA-1 upon antigen recognition by the T cell receptor [21]. This association facilitates Fyn protein tyrosine kinase mediated phosphorylation of Tyr^322^ of CD226 [21] and this might play an important role for Th1 polarization from naïve T cells since Th2 clones express low level of Fyn protein in mice and CD4+ naïve T-cells from Fyn-deficient mice polarize towards Th2 cells even in the absence of IL-4 and IL-13 [22], [23]. A more recent study has implicated CD226 as a signal transducer of LFA-1 following T cell activation [24]. Administration of CD226 monoclonal antibody inhibited expansion of PLP-139-151 specific CD4+ T-cells and delayed the onset as well as severity of EAE [18]. In addition, Shibuya et al. showed that a dominant negative mutation in the CD226 signaling domain in naïve human CD4+ T-cells strongly suppressed Th1 differentiation, suggesting that CD226 signaling is critical for Th1 differentiation [24]. Our findings provide evidence that CD226 is indeed up-regulated early on CD4+ T-cells, and we for the first time report the kinetics of CD226 expression on CD4+ T-cells in the blood of mice with EAE.

Another well-known and very important Th1 cell surface molecule, T-bet, is a transcription factor that was first associated with the differentiation of Th1 cells and IFN-γ production by CD4+ T cells [25]. Based on its ability to induce a pro-inflammatory immune response, T-bet has been found to regulate factors, including but not limited to STAT-1 expression and IL-23R transcription, which have been linked to pathogenicity in EAE and MS. T-bet is essential for the development of EAE, as T-bet deficient mice are resistant to EAE induction [26], [27], [28]. Myelin-specific T cells from T-bet-deficient mice fail to transfer disease to wild-type mice [27], [28], confirming that T-bet is necessary for the generation of encephalitogenic T cells. Moreover, up-regulation of T-bet expression has been reported during exacerbations in the peripheral blood CD4+ T-cells of RRMS patients compared to patients in remission and healthy controls [29].

Our results show that CD4+ T-cells in blood do significantly upregulate CD226 and T-bet expression in mice at EAE onset when compared to naïve mice that had very low detectable levels of CD226 and T-bet (Fig. 4). Furthermore, CD226 and T-bet expressing CD4+ T-cells remained elevated in the blood of control groups, but were significantly reduced after RTL551 treatment, thus indicating early RTL-mediated inhibition of CD4-T cells expressing these key pathogenic Th1 markers.

In EAE, D3 post onset was marked by a significant increase in CD44 expressing CD4+ T-cell in the blood and spleen of control groups of mice (Fig. 5). Increases in effector CD4+ T-cells were detectable as early as EAE onset, although the difference only became significant on D3 post onset. CD44 is up-regulated on naïve T cells after TCR mediated activation and its high expression is maintained on antigen experienced CD4+ T-cells [30]. While control groups of mice exhibited substantial percentages of CD4+ effector T-cells by D3, RTL551 treatment inhibited the maintenance of CD4+ effector T-cells in the periphery This was also associated with decrease in IL-17 and IFN-γ secreting cells in the spleen (Fig 6). Taken together, these results suggest that RTL551 treatment strongly reduced the Th1 immune response in the periphery early during EAE. No effects of empty RTL550 on Th1 and CD44 cell markers on CD4+ T-cells were noted, thus implicating MOG-35-55 peptide dependent effects of RTL551 on MOG-35-55 primed CD4+ T-cells. This result supports our earlier observations that cognate T-cell specificity is indispensable for successful RTL treatment effects [4].

We have previously reported that RTL treatment dramatically reduces CNS infiltration in mice when treatment is started at onset of EAE [3], [16]. In the current study, CNS tissue of mice with EAE was examined at early time points after RTL551 treatment in order to identify events initiated in the CNS that lead to subsequent resolution of inflammation (Fig. 7). Our results demonstrated rapid and progressive reductions in lesions and CNS inflammatory cell numbers during the treatment phase of EAE with RTL551, with significant changes observed as early as 1 day after a single injection of RTL551.

Two additional very dramatic and rapid changes induced in the CNS by a single RTL551 injection were: 1) selective reduction in absolute numbers of total and T-bet expressing CD4+ T-cells; and 2) significant down-regulation in IL-17 and IFN-γ secretion by MOG-35-55 reactive cells isolated from the CNS of RTL551 treated mice compared to control groups of mice. D1 post onset is a very active early phase of EAE, with CD4+ T-cells playing a crucial role as initiators of the disease. Indeed, at this time point the CNS cells from the control groups of mice had the greatest frequency of T-bet expressing CD4+ T-cells (Fig. 8) and secreted high levels of IL-17 and IFN-γ when cultured ex vivo with MOG peptide (Fig. 9A & B). Recent data have raised the possibility that T-bet's role in T cell differentiation is not limited to Th1 cells and therefore, lack of T-bet expressing CD4+ T-cells in the CNS of mice treated with RTL551 might attribute to the reduction of both IL-17 and IFN-γ. In support of this assertion, it has been shown that generation of autoreactive Th17 cells in the absence of T-bet is not sufficient to induce autoimmunity, even when Th17 cells are differentiated in vivo [26], [31]. Moreover, administration of T-bet siRNA to mice immunized with myelin peptides in CFA reduces both IFN-γ and IL-17 production [32]. Abrogation of IL-17 production in the CNS early during EAE could have important implications for reducing subsequent disease progression, best explained by a recent study that used a combination of microscopic techniques to discern the process of immune cell mediated neuronal damage [14]. This study emphasized that during EAE, the presence of CD4+ T-cells is the most relevant feature for immune mediated neuronal dysfunction and that CNS-specific Th17 cells play a dominant role compared to Th1 cells in initiation of neuronal injury [14]. Absence or reduction of IL-17 secreting cells in the CNS during early phases of EAE in RTL551 treated mice would abolish or mitigate the neurotoxic effects of this inflammatory cascade in the CNS and downstream recruitment of other inflammatory cells. While it is clear from our data that one RTL551 injection prevents the recruitment of additional CD4+ T-cells into the CNS, the mechanisms that limit infiltration of inflammatory cells remain an open question.

We thus hypothesize that RTL treatment of EAE is a two step process where binding of the truncated form of MHC Class II to mononuclear cells (MNC), including monocyte subpopulations, plays a critical role in initiating a tolerogenic response in CD4+ T-cells marked by specific ligation of the TCR and signaling events that result in the reduction of encephalitogenic CD4+ T-cells expressing T-bet and CD226 and secreting IL-17. In support of this proposed mechanism, we have shown previously that RTLs bind to antigen presenting cells in a peptide independent manner [5]. Furthermore, our recent data demonstrates that cell-bound RTL can be detected within the lumbar region of the spinal column for at least two days after completion of treatment of mice with EAE, suggesting potential interactions between RTL-bound APCs and encephalitogenic CD4+ T-cells not only in the periphery but also in the CNS. Future studies will be performed to confirm whether the reduction in CD4+ T-cells observed in blood and CNS is due to deletion of antigen primed CD4+ T-cells or their re-routing to other lymphoid organs besides spleen.

These novel results demonstrate that a major effect of RTL therapy is to attenuate Th1 specific changes in CD4+ T-cells during EAE and prevent expansion of both Th1 and Th17 effector T-cells that mediate clinical signs and CNS inflammation in EAE.
